# A Blockchain-Based Privacy Information Security Sharing Scheme in Industrial Internet of Things

**DOI:** 10.3390/s22093426

**Published:** 2022-04-30

**Authors:** Yue Wang, Tingyu Che, Xiaohu Zhao, Tao Zhou, Kai Zhang, Xiaofei Hu

**Affiliations:** 1 National and Local Joint Engineering Laboratory of Internet Applied Technology on Mines, China University of Mining and Technology, Xuzhou 221008, China; wyxz@cumt.edu.cn (Y.W.); tingyu@cumt.edu.cn (T.C.); kdstutaozhou@cumt.edu.cn (T.Z.); kaizhang@cumt.edu.cn (K.Z.); 2 School of Information and Control Engineering, China University of Mining and Technology, Xuzhou 221008, China; 3 School of Information and Business Management, Chengdu Neusoft University, Chengdu 611844, China; huxiaofei@nsu.edu.cn

**Keywords:** privacy information security sharing, Industrial Internet of Things, blockchain, consensus algorithm, incentive mechanism

## Abstract

Due to the competitive relationship among different smart factories, equipment manufacturers cannot integrate the private information of all smart factories to train the intelligent manufacturing equipment fault prediction model and improve the accuracy of intelligent manufacturing equipment fault detection. The use of a low fault recognition rate model for smart factories will cause additional losses for them. In this work, we propose a blockchain-based privacy information security sharing scheme in Industrial Internet of Things (IIoT) to solve the sharing problem of private information in smart factories. Firstly, we abstract smart factories as edge nodes and build decentralized, distributed trusted blockchain networks based on Ethereum clients on simulated edge devices and propose an Intelligent Elliptic Curve Digital Signature Algorithm (IECDSA) to guarantee the ownership of shared information by edge nodes. Secondly, we propose the Reputation-based Delegated Proof of Stake (RDPoS) consensus algorithm to improve the security and reliability of the Delegated Proof of Stake (DPoS) consensus algorithm. Furthermore, we design and implement an incentive mechanism based on information attributes to increase the motivation of edge nodes to share information. Finally, the proposed solution is simulated. Through theoretical and simulation experiments, it is proved that the blockchain-based privacy information security sharing scheme in IIoT can improve the enthusiasm of edge nodes to share information on the premise of ensuring the security of information sharing.

## 1. Introduction

With the continuous advancement of Industry 4.0 on a global scale, more and more intelligent manufacturing equipment is widely used in smart factories. Usually, the smart factory will push the computing resources of the cloud server to the edge of the network to build a cloud-side collaboration architecture to meet the requirements of intelligent manufacturing equipment for high computing power and low latency and ensure the stable production of the smart factory [1,2]. However, the failure of intelligent manufacturing equipment will bring unpredictable losses to smart factories. Intelligent manufacturing equipment manufacturers minimize the loss of smart factories by using predictive maintenance methods to issue failure warnings before products fail [3]. Intelligent equipment manufacturers use the data fusion theory of multi-intelligent manufacturing equipment data fusion to improve the accuracy of intelligent manufacturing equipment failure prediction models and reduce the false early warning information caused by the waste of maintenance personnel manpower [4]. However, for smart factories, when their products are manufactured, a large amount of private information about the product is stored in the intelligent manufacturing equipment. Smart factories will instinctively protect private information and will not easily share information, eventually forming an IIoT information island phenomenon [5,6].

Information sharing is one of the most effective ways to solve the phenomenon of information islands, and it is also very suitable for IIoT scenarios [7,8,9]. The security of the information sharing process is a thorny issue for private information. Traditional information sharing is mostly achieved through cloud data sharing. However, traditional cloud storage is faced with the problems of single point attack, transmission delay, and resource waste [10]. Blockchain technology, as a new type of distributed architecture technology, has been widely used, providing a new solution for information security sharing, and it can effectively solve the problems faced by traditional information sharing [11,12,13]. Privacy information is stored in the form of a Merkel tree after being multi-hashed in the blockchain [14]. When the privacy information stored in the blockchain is tampered with by the attacker, the hash value of the Merkle tree root will also be changed due to the private information, and other nodes in the blockchain environment will immediately detect the tampered data and ensure the consistency of the global data through the unique consensus mechanism of the blockchain [15]. The blockchain consensus mechanism is also one of the important factors affecting the secure sharing of privacy information. In addition, the speed of the consensus mechanism will also directly affect the speed of privacy information sharing [16].

As the earliest consensus algorithm, the Proof of Work (PoW) consensus algorithm was not only used to maintain the smooth operation of the Bitcoin blockchain network but also applied to the Ethereum client [17]. This algorithm not only wastes node resources when competing for bookkeeping rights but also affects the throughput of the blockchain network system. The Proof of Stake (PoS) consensus algorithm uses proof-of-stake, which not only reduces the waste of blockchain node resources caused by the PoW consensus algorithm to a certain extent but also improves the throughput of the blockchain network system [18]. However, the PoS consensus algorithm is prone to the risk of forking blockchains. The Delegated Proof of Stake (DPoS) consensus algorithm uses voting campaigns instead of mining in PoS consensus algorithms and PoW consensus algorithms, with low consumption, high throughput, and low latency [19]. However, the DPoS consensus algorithm not only lacks reward and punishment measures for blockchain nodes in the process of carrying out voting campaign operations but also risks malicious nodes being selected as proxy nodes to participate in the campaign. The Practical Byzantine Fault Tolerance (PBFT) consensus algorithm can achieve the same transaction processing speed as the DPoS consensus algorithm but is not suitable for blockchain networks with a large number of nodes [20]. At present, the consensus algorithm still has the problems of node scale, performance, and fault tolerance, which are difficult to balance.

In order to solve the above problems existing in the consensus algorithm and ensure the speed and reliability of private data sharing among smart factories, this work aims to promote the sharing of private information about intelligent manufacturing equipment in smart factories and provide privacy information for the training of intelligent manufacturing equipment failure prediction models. We propose a blockchain-based privacy information security sharing scheme in IIoT to ensure the secure sharing of privacy information among different smart factories. The main contributions of this work are as follows:

- The cloud-edge collaboration architecture of the smart factory is analyzed, and the edge-end network architecture based on edge servers is established. Then, the Intelligent Elliptic Curve Digital Signature Algorithm (IECDSA) is proposed to determine the ownership of the smart factory’s private information. In contrast to the traditional method, trusted storage and distribution of keys was implemented by the Key Distribution Smart Contract (KDSC), which reduces the risk of keys being tampered with and more securely guarantees the ownership of the shared private information by smart factories.
- The working principle of the DPoS consensus algorithm is analyzed, and in view of the situation that the malicious node is selected as a proxy node due to “hoarding” in the election process, the Reputation-based Delegated Proof-of-Stake consensus algorithm (RDPoS) is proposed. The algorithm performs a weighted operation on the number of node votes and reputation values and selects proxy nodes to participate in the consensus process according to the weighted operation results. Compared with the existing DPoS consensus algorithm, the probability of malicious nodes being selected as proxy nodes is reduced, and the security and reliability of the consensus reached between blockchain nodes are effectively improved.
- In view of the phenomenon that smart factories protect their own private information and refuse to participate in information sharing, a trusted incentive smart contract based on information attributes is constructed. Furthermore, a trusted network incentive environment without third party involvement is implemented, sending reward points to smart factories that provide private information sharing and ensuring the enthusiasm of smart factories in sharing information. Compared with the traditional incentive mechanism, the incentive mechanism realized by smart contracts is not interfered with by external factors, ensuring the fairness, impartiality, and openness of the incentive mechanism.

The rest of this work is organized as follows: Section 2 is the related work. Section 3 provides a detailed description of the proposed solution, including an overview of the overall solution, a network architecture, and a security analysis. Section 4 focuses on theories related to smart factory data ownership, blockchain data storage, the RDPoS consensus algorithm, and the incentive mechanisms based on information property. Section 5 provides an analytical discussion of the experimental results. Section 6 summarizes the full text.

## 2. Related Work

In [21], the authors propose a privacy-preserving data sharing framework for the Industrial Internet of Things that provides privacy protection for data contributors by interfering with the data provided by them. However, the framework does not take into account the enthusiasm of data contributors in contributing data. In [22], an asynchronous federated learning scheme is proposed that uses deep reinforcement learning (DRL) for node selection to improve efficiency, integrates machine learning models into the blockchain, and performs two-stage verification to ensure the reliability of shared data. The scheme also ignores the incentives of information providers to share data and malicious decisions in the process of the blockchain consensus mechanism [23].

The DPoS consensus algorithm is widely used due to its low energy consumption, high throughput, and dynamic scalability. However, the DPoS consensus algorithm suffers from the problems of malicious nodes being easily selected as proxy nodes and the low motivation of participating nodes to vote during the working process. In [24], in view of the low voting motivation of nodes and the lack of reference basis for nodes in the voting process, the concepts of token investment and side chains were introduced into the DPoS consensus algorithm, which effectively improved the voting motivation of nodes. However, the algorithm’s excessive reliance on tokens can easily lead to the emergence of malicious nodes. In [25], an improved ring-based coordinator election algorithm is proposed to optimize the election process in the DPoS consensus algorithm, which further improves the decentralization and fairness of the DPoS consensus algorithm, but the algorithm suffers from the risk of not reaching consensus among nodes. In [26], the introduction of node behavior monitoring and Borda count voting to select proxy nodes in the DPoS consensus algorithm reduces the probability of a malicious node being elected as a proxy node and improves the fairness of the election, although the scheme only considers the detection of the behavior of witness nodes generating blocks.

The incentive mechanism can improve the enthusiasm of smart factories to share private information, and the incentive mechanism can be roughly divided into two types: game-based incentives and external incentive-based incentives [27]. Kang et al. introduced reputation as an indicator of the reliability and trustworthiness of mobile devices in federated learning and proposed an effective incentive mechanism that combines reputation with contract theory to incentivize high-reputation mobile devices with high-quality data to participate in model learning, but the accuracy of reputation calculation in this scheme needs to be improved [28]. Zhan et al. studied the incentive mechanism of federated learning and designed an incentive mechanism based on deep reinforcement learning (DRL) to determine the optimal pricing strategy of the parameter server and the optimal training strategy of the edge node to motivate the edge node to contribute to the model training, but the method relies on the computing resources of the edge node [29]. In [30], an incentive mechanism for rational miners to purchase computing resources in the blockchain network environment of edge computing establishes a two-stage Stackelberg game model between miners and edge service providers (ESP) to maximize profits under two mining schemes, but this mechanism is not suitable for multiple ESP.

This work analyzes the above literature, and in view of the shortcomings of existing methods, proposes a blockchain-based privacy information security sharing scheme in IIoT to ensure the secure, fast, and active sharing of information among edge nodes (e.g., smart factories).

## 3. Scheme in Detail

This section provides a detailed description of the blockchain-based privacy information security sharing scheme in IIoT in terms of general scheme overview, system model, and security analysis.

### 3.1. The Overall Scheme

In this work, we abstract smart factories as edge nodes and build decentralized, distributed trusted blockchain networks based on Ethereum clients on simulated edge devices. Based on this architecture, a blockchain-based privacy information security sharing scheme in IIoT is proposed. In this scheme, the following steps are performed by the edge nodes of the information sender for information sharing.

Step 1. The information sender edge node uses the Intelligent Elliptic Curve Digital Signature Algorithm (IECDSA) to sign the information to be shared and stores the signed shared information in the blockchain.

Step 2. Information sharing among edge nodes is via the Reputation-based Delegated Proof of Stake (RDPoS) consensus algorithm.

Step 3. The received information is verified by the IECDSA at the edge node of the information receiver.

Step 4. The information receiver edge node provides rewards to the edge node of the information sender based on the incentive mechanism of information attribute to ensure the enthusiasm of the edge node in the network to share information.

**Figure 1:**
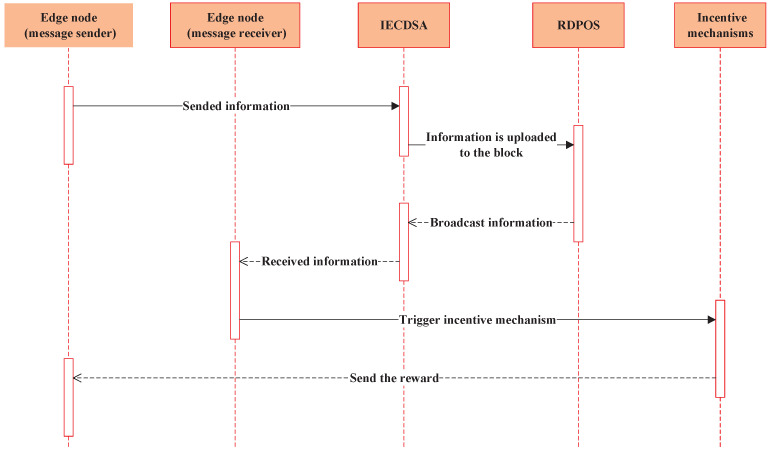
Scheme workflow.

The workflow of the blockchain-based privacy information security sharing scheme in IIoT is shown in Figure 1.

Sended information: The information sender edge node signs the information to be shared using IECDSA, declaring its ownership of the shared information.

Information is uploaded to the block: The information sender edge node uploads the signed message to the block in preparation for information sharing.

Broadcast information: The blockchain uses the RDPoS consensus algorithm to achieve consensus on the information stored in the block, enabling information sharing among edge nodes.

Received information: The information receiver edge node uses IECDSA to verify the identity of the information sender edge node.

Trigger incentive mechanism: After the information receiver edge node determines the identity of the information sender edge node, the incentive mechanism is triggered.

Send the reward: The triggered incentive mechanism provides a reward to the information sender edge node according to the pre-defined reward rules in the trusted incentive smart contract.

### 3.2. The Network Architecture

**Figure 2:**
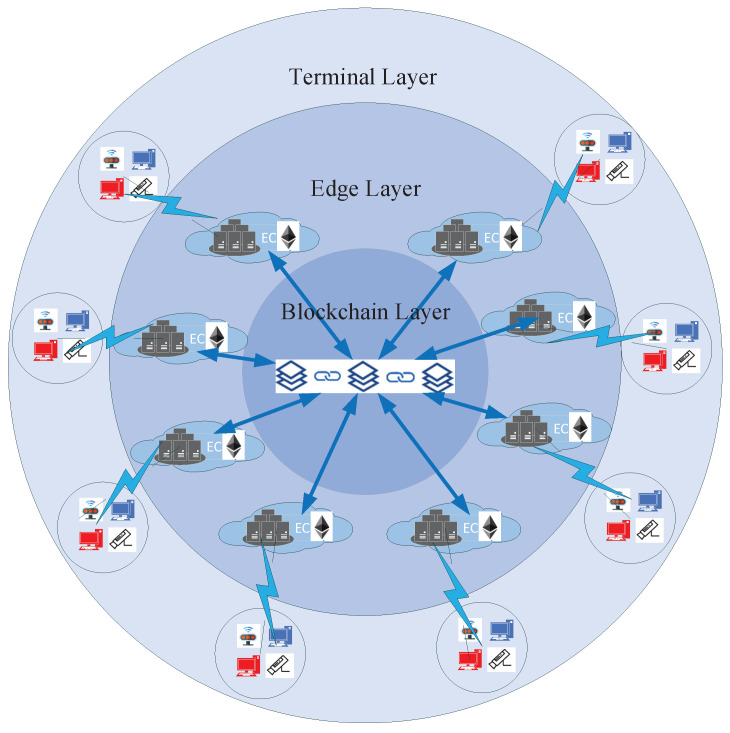
Network architecture.

As shown in Figure 2, the network architecture of the blockchain-based privacy information security sharing scheme in IIoT can be divided into three layers: the terminal layer, the edge layer, and the blockchain layer from the bottom up.

Terminal Layer: A group of terminal devices $TD=\{td_{1},td_{2},td_{3},\cdot \cdot \cdot ,td_{n}\}$ are connected to a high-performance edge device using the terminal device access technology. The edge device stores and processes the data of its subordinate terminal devices and provides services for the terminal devices.

Edge Layer: Divide the physical environment into different regions $R=\{r_{1},r_{2},r_{3},\cdots ,r_{n}\}$ by region and deploy a set of edge devices in different regions based on the load capacity of the edge devices $ED=\{ed_{1},ed_{2},ed_{3},\cdot \cdot \cdot ,ed_{n}\}$. These edge devices (edge nodes) are made up of edge layers $EL=\{r_{1_{ed_{1\cdot \cdot \cdot i}}},r_{2_{ed_{i+1\cdot \cdot \cdot k}}},r_{3_{ed_{k+1\cdot \cdot \cdot l}}},\cdot \cdot \cdot ,r_{n_{ed_{m\cdot \cdot \cdot n}}}\}$.

Blockchain Layer: At the edge layer, a blockchain network (blockchain layer) is formed by deploying an Ethereum client with a RDPoS consensus algorithm on high-performance edge devices. The consensus mechanism of the blockchain ensures the consistency of data among blockchain nodes (edge nodes).

### 3.3. Security Analysis

This section provides a security analysis of the blockchain-based privacy information security sharing scheme in IIoT.

#### 3.3.1. The Security of Information Storage

In our proposed scheme, we still use the Merkle tree of data storage structure used by the blockchain to store information. If the shared information stored in a block is tampered with, then the hash value of the Merkle tree root is changed, at which point the consensus mechanism of the blockchain will calculate the proportion of the shared information stored in the current block to the shared information stored in blocks of the same block height in all blockchain nodes (edge nodes). If the percentage is less than 51%, the information has been tampered with. In this case, the RDPoS consensus algorithm will overwrite the tampered information with the correct information to ensure that the data are consistent among the edge nodes. If the attacker wants to make the ratio exceed 51%, he needs to control 51% of the edge nodes in the network to achieve this, and it is not realistic for the attacker to control 51% of the edge nodes at the same time in a short period of time.

#### 3.3.2. The Security of Information Sharing

We have improved the problems with the widely used Delegated Proof of Stake consensus algorithm, which suffers from the problems of malicious nodes being easily selected as proxy nodes and the low motivation of nodes involved in voting and propose a Reputation-based Delegated Proof of Stake (RDPoS) consensus algorithm. The RDPoS consensus algorithm first supervises the behavior of nodes through a reputation model and assigns corresponding behavior scores according to the normality of nodes’ historical behavior, calculates the reputation value and trustworthiness status of nodes, and finally selects proxy nodes to participate in the consensus process. In addition, the algorithm also designs a hybrid mechanism model to ensure the motivation of nodes to participate in voting. The RDPoS consensus algorithm not only guarantees the security of the proxy node election process but also ensures the motivation of participating voting nodes, and it improves the security of the consensus process.

#### 3.3.3. The Fairness of Information Sharing

In our proposed scheme, an incentive mechanism based on information properties is designed, which is implemented by a trusted incentive smart contract. The trusted incentive smart contract can be executed automatically without the involvement of a third party. In a blockchain environment, smart contracts can only be changed through version replacement, and if an edge node wants to modify the sharing rules inside a trusted incentive smart contract to give itself additional revenue, it needs to redeploy the contract to do so. However, this process is open and transparent and monitored by all edge nodes. In addition, we use an incentive mechanism based on information attributes, where the information receiver edge nodes can provide rewards to the information sender edge nodes based on the value of the shared information for their own use, reducing the waste of edge node assets to a certain extent. Therefore, our scheme is therefore fair and frugal.

In summary, we analyzed the security of our proposed scheme from three aspects: information storage security, information sharing security, and information sharing fairness, and the results proved that our proposed blockchain-based privacy information security sharing scheme in IIoT is safe and reliable.

## 4. Methods

In this section, the Intelligent Elliptic Curve Digital Signature Algorithm (IECDSA), the Reputation-based Delegated Proof-of-Stake consensus algorithm (RDPoS), and the incentive mechanism based on information attributes are implemented separately.

### 4.1. Intelligent Elliptic Curve Digital Signature Algorithm (IECDSA)

Our proposed scheme uses the Intelligent Elliptic Curve Digital Signature Algorithm to ensure ownership of shared information by edge nodes, as follows.

Generator G(x,y) is used as a public parameter on the elliptic curve $E_{p}\left(a,b\right)$. We chose PrK as the private key for the digital signature in $E_{p}\left(a,b\right)$. The public key can then be expressed as:

(1) PuK=PrK*G

The message sender edge node signs the message *m* to be shared (the signature consists of two parts s_b and s_b).

The pseudo-random numbers are generated using the linear congruence algorithm, as shown in Equation (2) [31].

(2) $$RandSeed=\left(a*RandSeed+c\right)\%m$$

where a,c,m are the constants set by the generator.

Point multiplication of RandSeed with the generator G(x,y) to the point *P*.

(3) $$P=RandSeedG\left(x,y\right)=\left(x_{1},y_{1}\right)$$

The s_a part of the signature is generated by performing the operation according to Equation (4) using the horizontal coordinates of the point $P(x_{1},y_{1})$ and the prime number *n*.

(4) $$s\_a=x_{1}\;\mathrm{mod}\;n$$

Calculate the hash value of the shared information by Equation (5).

(5) h=Hash(m)

The signature information is obtained by Equation (6).

(6) $$s\_b=RandSeed^{-1}\left(h+PrKs\_a\right)\;\mathrm{mod}\;n$$

where PrK is the private key, *h* is the hash of the shared information, s_a is the signature information, RandSeed is a random number, and *n* is a prime number.

The message sender edge node’s signature information for shared messages is (s_a,s_b).

We designed the key distribution smart contract as shown in Algorithm 1 to enable intelligent, supervised, and secure distribution of public keys among edge nodes without the involvement of third parties. When the information sender edge node sends the shared information, the public key is stored in the blockchain through the key distribution smart contract, and the storage structure and procedure of the block in the blockchain are described in Appendix A.

After receiving the shared information, the information receiver edge node uses the public key to verify the signature information of the message sender. The information receiver edge node verifies that s_a and s_a are integers in $\left[1,n-1\right]$; then, it computes the hash *h* of the shared information according to Equation (5), followed by the value of $w,u_{1},u_{2}$.

(7) $$w=s\_b^{-1}\;\mathrm{mod}\;n$$

(8) $$u_{1}=hw\;\mathrm{mod}\;n$$

(9) $$u_{2}=s\_a^{-1}w\;\mathrm{mod}\;n$$

**Algorithm 1** Key Distribution Smart Contract (KDSC)
**Input:**PuK, EList // PuK is public key. EList is information set of edge nodes in the network.
**Output:** State of public key distribution.
1:KdList // This list is used to store the public key distribution status.2:**for** i to size(EList) **do**3:    PuK→EList(i) // Assigns public keys to edge nodes in the network.4:    KdList.Add(Stae(EList(i)) // Stae(EList(i)) is the status in which the current edge node distributes the public key.5:
**end for**
6:
**return** KdList




Bringing the parameters $u_{1},u_{2},G\left(x,y\right)$ and the public key PuK into Equation (10) yields the point *X*.

(10) $$X=u_{1}G+u_{2}PuK=\left(x_{1},y_{1}\right)$$

Take the horizontal coordinate $x_{1}$ of point *X* and prime number *n* for modular arithmetic according to Equation (11). If the equation is true, the signature is valid; otherwise, the signature is invalid.

(11) $$x_{1}\;\mathrm{mod}\;n=s\_a$$

The IECDSA is not only resistant to plaintext attacks but also to ciphertext attacks, so that even if an attacker intercepts the signature message, he cannot forge a valid signature message. The IECDSA is shown in Algorithm 2.
**Algorithm 2** Intelligent Elliptic Curve Digital Signature Algorithm (IECDSA)**Input:**MList, EList // MList is the information shared set by the edge nodes of the message sender. EList is information set of edge nodes in the network.**Output:** Status of signatures and verification of signatures.1:**for** i to size(MList) **do**2:    Selecting the data signature private key.3:    PuK=PrK*G // Calculating a digitally signed public key. distribution the public key.4:    $RandSeed=\left(a*RandSeed+c\right)\%m$ // Generate random numbers.5:    $P=RandSeedG\left(x,y\right)=\left(x_{1},y_{1}\right)$ // Calculation of the parameter *P*.6:    Generate data signature s_a by Equation (4).7:    h(MList(i))=Hash(MList(i)) //Calculating hash values of shared information.8:    Generate data signature s_b by Equation (6).9:    ***KDSC(PuK,EList)*** // Key Distribution Smart Contracts enables intelligent distribution of public keys.10:    **if** s_a&&s_b∉[1,n−1] **then** // Information receiver edge nodes verify signatures.11:        Signature verification failure.12:    **else**13:        Calculating hash values of shared information by Equation (5).14:        Calculate the parameters $w,u_{1},u_{2}$ according to Equations (7)–(9).15:        $X=u_{1}G+u_{2}PuK=\left(x_{1},y_{1}\right)$ // Calculate the parameter *X*.16:        **if** $x_{1}\;\mathrm{mod}\;n=s\_a$ **then**17:           Successful signature verification.18:        **else**19:           Signature verification failure.20:        **end if**21:    **end if**22:**end for**23:**return** Status of signatures and verification of signatures.

### 4.2. Reputation-Based Delegated Proof of Stake (RDPoS)

The process of the Reputation-based Delegated Proof of Stake consensus algorithm is as follows. Firstly, the node’s behavior is monitored by the reputation model and assigned a corresponding behavior score; then, the node’s reputation value is calculated and the node’s trustworthiness status is defined by the reputation value, and finally the proxy node is selected to participate in the consensus process based on the reputation value and the number of votes. In the RDPoS consensus algorithm, blockchain nodes are divided into three categories: normal nodes, candidate nodes (voting nodes), and proxy nodes.

In the reputation model, a node is evaluated for trustworthiness based on its performance throughout the period and it is assigned a reputation value (*R*) to indicate the trustworthiness of the node. Assuming that *R* is a real number between 0 and 1, the larger the value of *R*, the higher the trustworthiness of the node. When new nodes join the blockchain network, the reputation value defaults to 0.5.

All acts behavior_j of the *i*-th node node_i in the period *T* are denoted as:

(12) $$behavior\_j=\{B_{1},B_{2},B_{j},...,B_{n}\}$$

where $B_{j}$ is the score of the *j*-th act of behavior_j and *n* is the number of acts.

Assuming that $B_{i(j)}$ denotes the behavior value of the *j*-th behavior of node node_i during the period *T*, the (j+1)-th behavior value $B_{i(j+1)}$ of node node_i is determined according to the type of node node_i (agent node and voting node). The rules for calculating the behavior value of a node are shown in Table 1.

The time interval for scoring the behavior of a node is the period *T*, and when the period ends the behavior value of node node_i is calculated according to Equation (13).

(13) $$B_{i}=\sum_{j=1}^{n}(B_{i(j)})$$

Calculate the reputation value of node node_i using the behavior value of node node_i.

(14) $$R_{T}^{node\_i}=\frac{1}{1+e^{-\varphi B_{i}}}$$

where *T* is the current period and φ is an adjustable parameter.

The Reputation Model Algorithm is shown in Algorithm 3.

To reduce the probability of a malicious node being selected as a proxy node, we classify the nodes into trusted status (e.g., Good, Normal, Abnormal and Error) by their reputation values. The node status corresponding to the reputation value, the weight of the reputation value and the weight of the number of votes are shown in Table 2.

When an edge node first joins the blockchain network, its default status is Normal, its reputation value is initialized to 0.5, and it is in the preferred position for subsequent participation in the campaign process. When an edge node has a good record, a block is generated and validated according to expectations, and as its reputation value gradually rises and will exceed threshold *a*, the node status switches to Good and the node in Good has a greater chance of being selected as a proxy node. Furthermore, in the second category, it will be selected in subsequent participation in the campaign process. If an edge node has generated incorrect records, validated invalid blocks and other irregularities, when its reputation value gradually drops below 0.5, the blockchain network converts the status of this edge node to Abnormal, and it is in the third category to be selected in the subsequent participation in the campaign process. If an edge node consistently generates invalid blocks or has persistent irregularities, when the reputation value of the edge node drops below *b*, the node’s status will switch to Error and it will be in the last category of the selected positions in the subsequent participation in the campaign process.
**Algorithm 3** Reputation Model Algorithm (RMA)**Input:** node node_i, penalty coefficient *x*, incentive increase factor *y*.**Output:**$R_{node\_i}$. // The reputation value of node node_i is $R_{node\_i}$1:$R_{node\_i}=0.5$ // Initialize the node reputation value.2:$B_{i}$ // The sum of the historical behavior of the node node_i.3:$B_{i(j)}=0$ // Initialize the behavioral score of node node_i.4:**if**node_i is a non-proxy node **then**5:    **if** node_i active participation in voting **then**6:        $B_{i\left(j+1\right)}=min\left(1,\left(1+y\right)B_{i\left(j\right)}\right)$7:        $B_{i}=B_{i}+B_{i\left(j+1\right)}$8:    **end if**9:    **if** node_i inactive participation in voting **then**10:        $B_{i\left(j+1\right)}=xB_{i\left(j\right)}$11:        $B_{i}=B_{i}+B_{i\left(j+1\right)}$12:    **end if**13:    **if** node_i cast an invalid vote **then**14:        $B_{i\left(j+1\right)}=0$15:        $B_{i}=B_{i}+B_{i\left(j+1\right)}$16:    **end if**17:**end if**18:**if**node_i is a proxy node **then**19:    **for** t=0 to *T***do** // *t* is the time in the cycle and *T* is the whole cycle time.20:        **if** node_i generates blocks and uploads them to the blockchain **then**21:           $B_{i\left(j+1\right)}=min\left(1,\left(1+y\right)B_{i\left(j\right)}\right)$22:        **end if**23:        **if** node_i did not generate the block on time **then**24:           $B_{i\left(j+1\right)}=xB_{i\left(j\right)}$25:           $B_{i}=B_{i}+B_{i\left(j+1\right)}$26:        **end if**27:        **if** node_i generates invalid blocks **then**28:           $B_{i\left(j+1\right)}=0$29:           $B_{i}=B_{i}+B_{i\left(j+1\right)}$30:        **end if**31:    **end for**32:**end if**33:$R_{node\_i}=\frac{1}{1+e^{-\varphi B_{i}}}$ // Calculate the reputation value of node node_i.34:**return**$R_{node\_i}$.

The weighting of reputation values and the weighting of the number of votes in Table 2 are used by Equation (15) to calculate the node scores.

(15) $$score_{i}=W_{1}R_{i}+W_{2}V_{i}$$

where $R_{i}$ is the reputation value of node node_i and $V_{i}$ is the total number of votes received by node node_i.

Ultimately, the edge nodes are ranked according to their scores, and the fixed number of nodes with the highest ranking are selected as proxy nodes to participate in block generation and verification.

The Reputation-based Delegated Proof of Stake Algorithm is shown in Algorithm 4.
**Algorithm 4** Reputation-Based Delegated Proof of Stake Algorithm (RDPoS)**Input:** Hash value of the current block.**Output:** Status of the block on the chain.1:DList. // The list of proxy nodes.2:NList. // The list of blockchain network nodes.3:RList. // The list of node reputation.4:VList. // The list of total node votes.5:Initialize the number of agent nodes to *N*.6:Flag = False. // Block out status default failure.7:**for**i=0 to len(NList) **do**8:    Use ***RMA*(NList[i])** to calculate the node reputation value *R*.9:    RList.Add(*R*)10:    Calculate the total number of node votes *V*.11:    VList.Add(*V*)12:**end for**13:Select a proxy node list DList.14:**for**t=0 to *T***do** // *t* is the time in the cycle and *T* is the whole cycle time.15:    Proxy nodes take turns generating blocks.16:    **if** Other nodes validated successfully **then**17:        Proxy nodes to upload blocks to the chain.18:        Flag = True19:    **end if**20:**end for**21:**return** Flag

In the DPoS consensus algorithm, there is the problem that only the proxy node that generates the block is rewarded and no other proxy nodes are rewarded. In addition, there are cases where both malicious and normal nodes receive the same reward when generating blocks, which undermines the original fairness of the blockchain. Our scheme combines a transaction fee incentive with a reputation incentive to propose a hybrid incentive mechanism to ensure the fairness of the RDPoS consensus algorithm. The transaction fee reward obtained by the node successfully generating the block is calculated according to Equation (16).

(16) $$\Delta F=\left\{\begin{array}{c} F+\frac{R_{node\_i}}{\sum_{j=1}^{j=N}R_{j}}*F,\;Node\;status\;is\;Good.\\ F,\;Node\;status\;is\;Normal.\\ F-\frac{R_{node\_i}}{\sum_{j=1}^{j=N}R_{j}}*F,\;Node\;status\;is\;Abnormal.\\ 0,\;Node\;status\;is\;Error. \end{array}\right.$$

where ΔF is the transaction reward for successfully generated block, $R_{node\_i}$ is the current reputation of the node node_i, *N* is the total number of proxy nodes in the blockchain network, and *F* is the transaction fee reward allocated for a successfully generated block in the blockchain network.

### 4.3. Incentive Mechanism Based on Information Attributes

When the edge nodes share information, the negative situation of information sharing may appear due to some subjective factors, which affects the enthusiasm of information sharing in the whole edge network. In order to address the above phenomenon, we propose an incentive mechanism based on information attributed in the scheme to ensure the motivation of information sharing among the edge nodes in the network.

One must add the properties for shared information before sending the message by the information sender edge node.

(17) $$m\to \{m_{type},m_{quantity},m_{limitation\;},m_{expected},m_{real}\}$$

where $m_{type}$ is the type of shared data, $m_{quantity}$ is the number of shared messages, $m_{limitation\;}$ is the timeliness of the shared messages, $m_{expected}$ is the reward expected by the information sender edge node, and $m_{real}$ is the real reward provided by the information receiver edge node for the information sender edge node.

The information sender edge nodes expect the following rewards.

(18) $$m_{expected}=\sum_{i=1}^{n}m_{expected_{i}}$$

where *i* is the number of information receiver edge nodes.

The demand for shared information is different for different types of information receiver edge nodes. We assign different weights to the attributes of the information based on the demand for shared information by information receiver edge nodes.

(19) $$\begin{array}{c} m_{real}=\sum_{i=1}^{n}w_{type_{i}}m_{type}+w_{quantity_{i}}m_{quantity}\\ +\sum_{i=1}^{n}w_{limitation{\;}_{i}}m_{limitation\;} \end{array}$$

where $w_{type_{i}}+w_{quantity_{i}}+w_{limitation{\;}_{i}}=1$, $w_{type_{i}}$ is the weight of the *i*-th information receiver edge node on the type of shared information, $w_{quantity_{i}}$ is the weight of the *i*-th information receiver edge node on the amount of shared information, and $w_{limitation{\;}_{i}}$ is the weight of the *i*-th information receiver edge node on the timeliness of shared information.

If the expected values of the information sender edge node and the information receiver edge node satisfy Equation (20), it means that the information sharing reward is provided successfully; otherwise, the information receiver edge node dynamically adjusts the proportion among the weights of the shared information attributes so that they satisfy Equation (20).

(20) $$m_{expected}-m_{real}<0.3$$

We design trustworthy incentive smart contracts to process real and trustworthy incentive transaction information in the blockchain to achieve a trustworthy incentive network environment without third party participation, ensuring fair, open, and transparent incentive distribution among edge nodes.

## 5. Simulation Experiments

We simulated 15 edge nodes using VMware Workstation running 15 Ubuntu 19.04 virtual machines on 5 Windows 10 machines. All edge nodes have the same configuration: Intel(R) Core (TM) i5-8250U processor at 2.13 GHZ and 2G of RAM. At the same time, a private blockchain network based on both the DPoS consensus algorithm for the Ethereum client and the RDPoS consensus algorithm for the Ethereum client on each virtual machine.

### 5.1. The Experiments of IECDSA

We tested the Digital Signature Algorithm (DSA), the RSA digital signature algorithm, and the IECDSA separately using the Bot-IoT Dataset collected by Koroniotis et al. [32].

**Figure 3:**
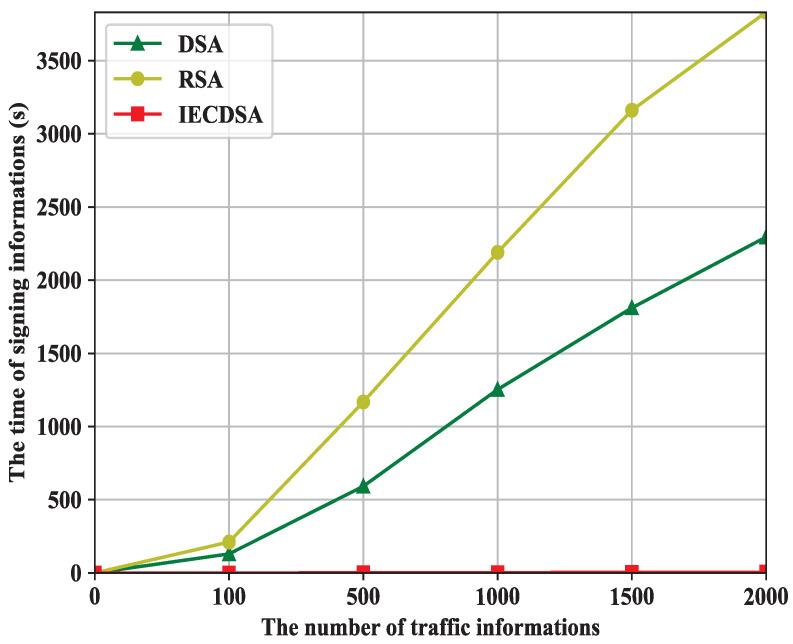
The time to sign information.

As shown in Figure 3, the DSA, RSA, and IECDSA for digital signature time consumption all show an increasing trend as the traffic information increases. The RSA is used to sign traffic information, consuming 130 s when the number of traffic information points reaches 100 and up to 2295 s when the number of traffic information points reaches 2000. The DSA is used to sign traffic information, consuming 211 s when the number of traffic information points reaches 100, and up to 3831 s when the number traffic information points reaches 2000. The IECDSA is used to sign traffic information, consuming 0 s when the number of traffic information points is 100 and only 5 s when the number of traffic information points reaches 2000. In general, the DSA consumes the most time signing traffic information and the IECDSA consumes the least time signing traffic information.

**Figure 4:**
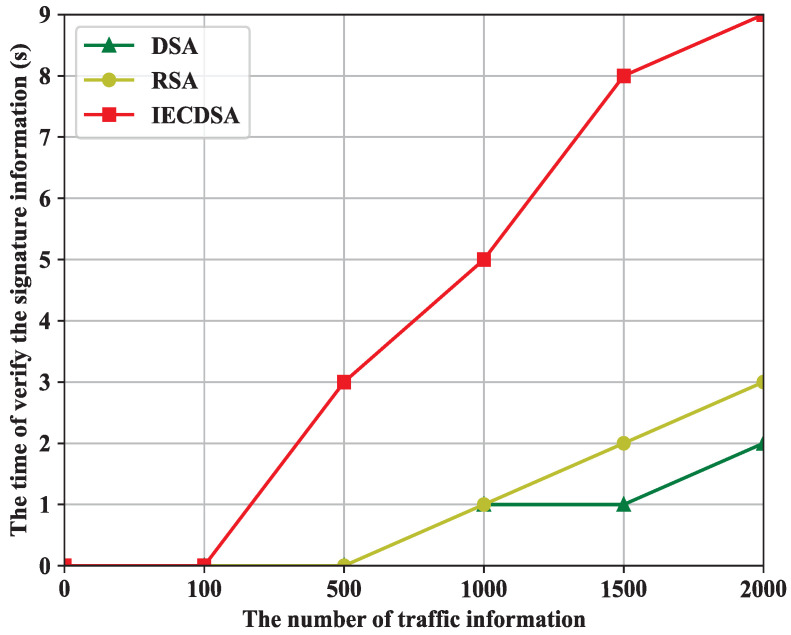
The time to verify the signature information.

As shown in Figure 4, the DSA, RSA, and IECDSA algorithms all show an increasing trend in time spent on digital signature verification as the traffic information increases. The RSA is used to verify the traffic information signature, consuming 0 s when the number of traffic information points is 100 and 2 s when the number of traffic information points reaches 2000. The DSA is used to verify the traffic information signature, consuming 0 s when the number of traffic information points is 100 and 3 s when the number of traffic information points reaches 2000. The IECDSA is used to verify the traffic information signature, consuming 0 s when the number of traffic information points is 100 and 9 s when the number of traffic information points reaches 2000.

In summary, the time taken by the three digital signature algorithms to sign and verify traffic information shows that the IECDSA algorithm has a huge advantage when it comes to digital signatures. Although the IECDSA algorithm takes relatively more time to verify the signature, the difference is within a few seconds. Hence, we proposed an intelligent elliptic curve digital signature algorithm which is more advantageous when processing information shared by edge nodes.

### 5.2. The Experiments of RDPoS

This section analyses the rationality of proxy node selection and the RDPoS consensus algorithm.

**Figure 5:**
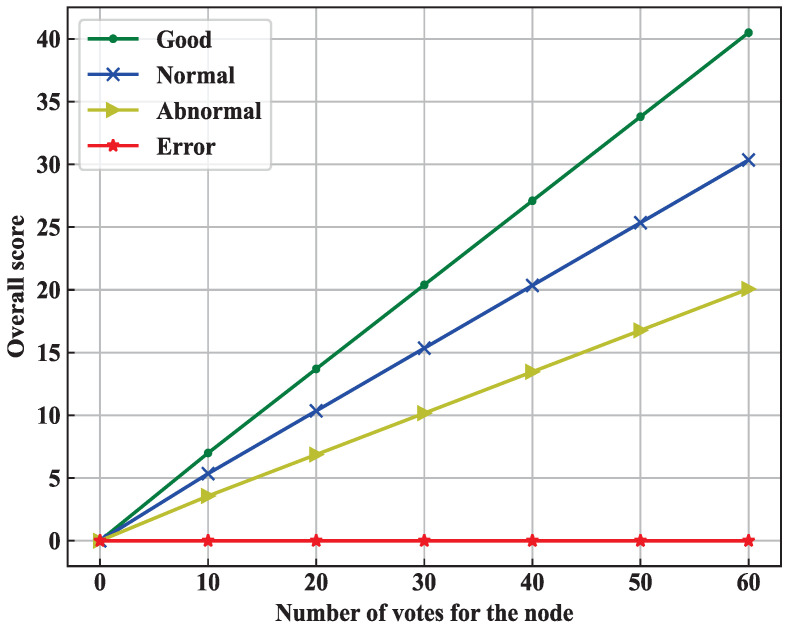
Changes in nodes with different reputation states.

Rationalization of proxy node selection: We chose four edge nodes with different reputation values for experimental validation: *Edge Node 1* (reputation value 0.8, trusted status Good), *Edge Node 2* (reputation value 0.6, trusted status Normal), *Edge Node 3* (reputation value 0.3, trusted status Abnormal), and *Edge Node 4* (reputation value 0.1, trusted status Error). The overall score corresponding to edge nodes receiving 10, 20, 30, 40, 50, and 60 votes was analyzed. The node status change is shown in Figure 5.

The nodes with *Good* status have an increasing rating as the number of votes increases, and the nodes with *Good* status are always ahead of the nodes with *Normal*, *Abnormal*, and *Error* status. The nodes with the status *Normal* have an increasing rating as the number of votes increases, and nodes with the status *Normal* are always ahead of those with the status *Abnormal* and those with the status *Error*. The nodes with *Abnormal* status have an increasing rating as the number of votes increases, and the nodes with *Abnormal* status have a higher rating than the node with *Error* status. The nodes with the status *Error* have a constant score of 0 as the number of votes increases. As the number of votes continues to increase, nodes with high reputation values consistently score ahead of other nodes, and in general, nodes with high reputation values are more likely to be selected as proxy nodes.

RDPoS consensus algorithm: We select four edge nodes as proxy nodes out of the 15 simulated edge nodes and use a random generator to vote against the others to ensure that the voting is closer to the real voting scenario. The number of votes received by each edge node after the 1st round of voting is shown in Table 3. The four edge nodes with the highest number of votes were selected as proxy nodes for consensus based on the voting results.

**Figure 6:**
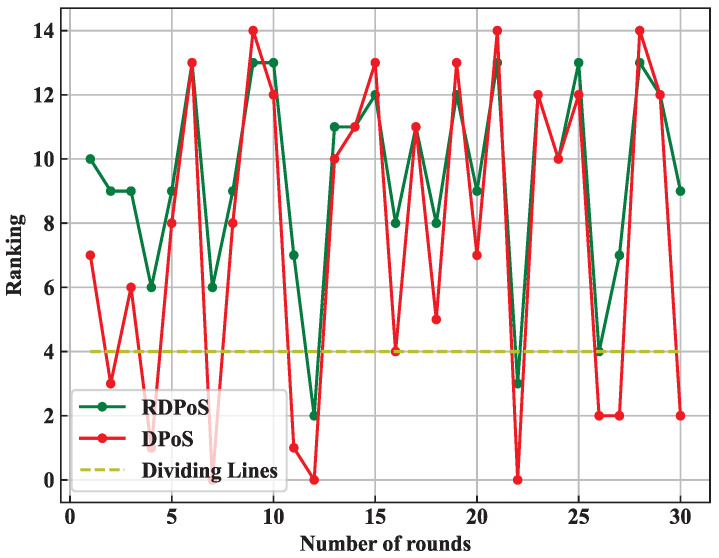
Ranking of anomalous *edge node C* per round.

Round 2 voting sets *edge node C* to *abnormal* status and the other edge nodes to *normal* status. The 2nd round of voting is repeated for 30 rounds by voting on the basis of the end of the 1st round of consensus. Figure 6 shows the ranking of *edge node C* among the candidate nodes after voting by the DPoS consensus algorithm and the RDPoS consensus algorithm, respectively.

From the ranking of *edge node C* in the 30 rounds of voting results, in the DPoS consensus algorithm, *edge node C* was selected as a proxy node for consensus 10 times, and the probability of an anomalous node being selected as a proxy node was 33.33%; in the RDPoS consensus algorithm, *edge node C* was selected as a proxy node for consensus only three times, and the probability of an anomalous node being selected as a proxy node was 10.00%.

The RDPoS consensus algorithm effectively reduces the probability of malicious nodes being selected in the process of selecting proxy nodes to ensure the security of the consensus.

### 5.3. The Experiments of Incentive Mechanism

In this section, we analyze trusted incentive smart contracts and incentive mechanisms based on information properties.

**Figure 7:**
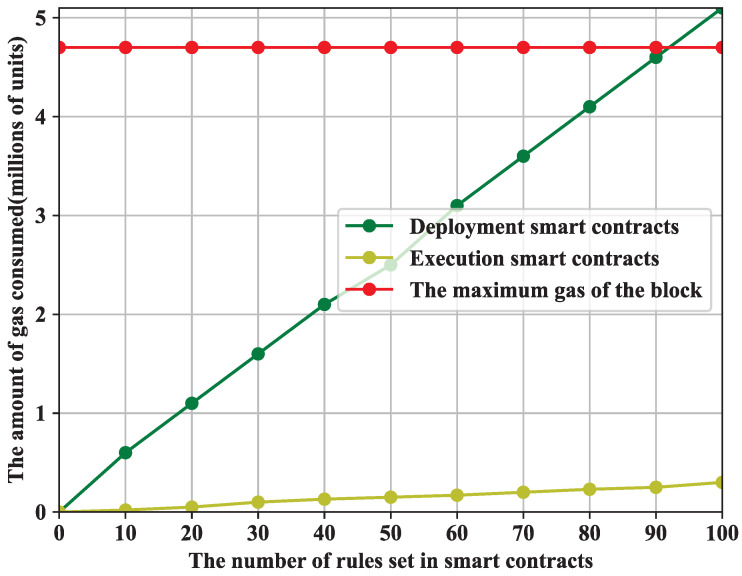
Smart contract gas consumption.

Trusted incentive smart contracts: Gas is finite for the users who need to consume it to send transactions and to deploy smart contracts and execute them. As shown in Figure 7, the deployment and execution costs of smart contracts vary linearly with the increasing number of set rules in the smart contract. The cost of deploying a smart contract increases as the number of rules in the contract increases, and the cost of executing a smart contract increases as the number of rules in the contract increases. Because smart contracts only need to be deployed once (paying for gas once on deployment) before they can be used, and they need to pay for gas every time they are executed, we see in the experimental results that their deployment cost is much higher than their execution cost. The red line in Figure 7 is the block of maximum gas, which is set when the Genesis block is initialized. It represents the maximum gas that a user is willing to pay to perform an operation or confirm a transaction, and if the block maximum gas is exceeded, the block will be rejected by the network.

Incentive mechanisms based on information property: We tested the proposed incentive mechanism based on information properties by deploying trusted incentive smart contracts in a simulated private blockchain network based on the RDPoS consensus algorithm for the Ethereum client, and the test results are shown in Table 4.

The experimental results show that 12 of the 15 simulated edge nodes with an incentive mechanism based on information attributes share more information than that shared in a normal case. The amount of information shared by *edge node E*, *edge node I*, and *edge node O* is slightly lower than the amount of information shared in a normal case. On the whole, the number of edge nodes sharing information under the incentive mechanism based on information attributes is significantly higher than the amount of information shared without the incentive mechanism. Thus, our proposed incentive mechanism based on information attributes stimulated the edge nodes to share information.

Although the number of simulated edge nodes during the experiment is limited, the above experimental results show that the blockchain-based privacy information security sharing scheme in the IIoT proposed in this work ensures the enthusiasm of smart factories in sharing private information under the premise of ensuring the security of private data.

## 6. Conclusions

In this work, we propose a blockchain-based privacy information security sharing scheme in IIoT to improve the motivation of smart factories to share information while ensuring the security of information sharing. Firstly, we propose an Intelligent Elliptic Curve Digital Signature Algorithm to sign the information shared by the smart factory and determine the ownership of the shared information. The algorithm not only protects the security of the key but also outperforms similar signature algorithms in terms of speed. Then, we propose a Reputation-based Delegated Proof-of-Stake consensus algorithm, which reduces the probability of malicious nodes being selected as proxy nodes and improves the security of data consistency among smart factories. Finally, we propose an incentive mechanism based on information attributes, and the amount of information shared by smart factories is significantly improved under the condition of using this incentive mechanism.

The scheme presented in this article was tested by the VMware Workstation, which affects the experimental results to a certain extent. In future work, we should test the proposed solutions on a real local area network. Although the incentive mechanism based on information attributes promotes the sharing of private data between smart factories to a certain extent, it ignores the competitive relationship among smart factories, and the introduction of game theory could be considered to improve the incentive mechanism in the future. 

## Figures and Tables

**Table 1:** The rules for calculating the behavior value of a node.

Value of Behavior	Voting Node	Agent Node
$min\left(1,\left(1+y\right)B_{i\left(j\right)}\right)$ ^1^	Voting active	Generate blocks and upload them to the blockchain
$xB_{i\left(j\right)}$ ^2^	Voting inactivity	Block not generated on time
0	Vote invalid	Generate invalid blocks

^1^ Where 0 < *y* < 0.03; ^2^ Where 0 < *x* < 1.

**Table 2:** Parameters corresponding to the status of the node.

Trusted Status	Reputation Value (*R*)	Weight of the *R* ($w_{1}$)	Weight of the Number of Votes ($w_{2}$)
Good	[a, 1] ^1^	[0.3, 0.5)	(0.5, 0.7]
Normal	[0.5, a)	0.5	0.5
Abnormal	[b, 0.5) ^2^	(0.5, 0.7]	[0.3, 0.5)
Error	R<b	0	0

^1^ Where 0.5 < a < 1; ^2^ Where 0 < b < 0.5. a, b represent thresholds, respectively.

**Table 3:** Results of the 1st round of voting.

Account	Edge Node	Reputation Value	Node Statu	Number of Vote
0x511⋯6202c7	A	0.5	Normal	13
0xbc3⋯5a7abd	B	0.5	Normal	4
0x61f⋯f6b828	C	0.5	Normal	22
0x59c⋯976260	D	0.5	Normal	21
0xd88⋯b33a21	E	0.5	Normal	23
0xf7a⋯0c927e	F	0.5	Normal	0
0xefa⋯d2eb37	G	0.5	Normal	3
0x23d⋯0cb24f	H	0.5	Normal	0
0x929⋯7dacef	I	0.5	Normal	6
0x640⋯e0576b	J	0.5	Normal	2
0x973⋯9d023	K	0.5	Normal	0
0xdd9⋯b1364	L	0.5	Normal	6
0x72c⋯a8c5b	M	0.5	Normal	0
0xfff⋯771332	N	0.5	Normal	4
0x5aa⋯e645cc	O	0.5	Normal	0

**Table 4:** Edge node information sharing results.

Edge Nodes	Amount of Information Shared	
	Incentive Mechanisms	No Incentive Mechanisms
A	453	200
B	502	321
C	433	365
D	625	432
E	425	430
F	335	332
G	249	230
H	587	438
I	442	445
J	443	246
K	332	296
L	629	516
M	587	540
N	368	352
O	321	332

**Figure A1:**
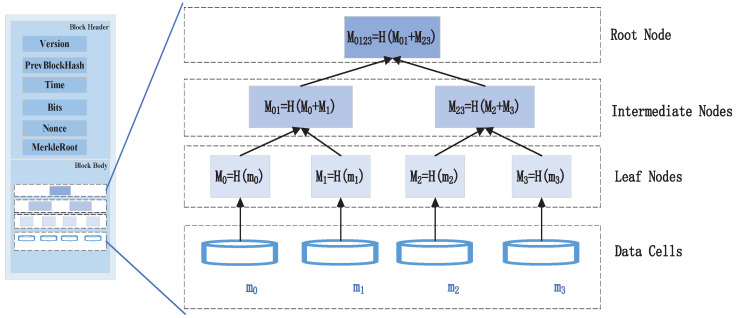
The storage structure of block.

## Data Availability

Not applicable.

## Appendix A. The Data Storage of Block

The block storage structure is shown in Figure A1. The block is divided into two parts: the block header, which consists of three sets of metadata: index data, consensus data, and transaction data, and the block body, which stores information shared by edge nodes in the form of the Merkle tree.

The Merkle tree is generated by Equation (A1).

(A1) $$Y=Hash(x_{i},x_{j})$$

where the length of *Y* is fixed. If one of the values of $x_{i}$ and $x_{j}$ changes, then the value of *Y* will also change.

Equation (A1) guarantees that any combination of two inputs will have a unique output value corresponding to it. It is impossible for an attacker to invert the values of $x_{i}$ and $x_{j}$ based on the value of *Y*.

The Merkle tree is divided from the bottom to the top into leaf nodes, intermediate nodes, and root nodes.

Leaf nodes: The hash value of the leaf node is obtained by hashing the data cell as a parameter. If the data block to be processed is an odd number, the last data cell needs to be copied so that the Merkle tree always remains a full Merkle tree.

(A2) $$M_{i}=H\left(m_{i}\right)$$

Intermediate nodes: The hash of an intermediate node is the hash of the sum of its two child node hashes, calculated by Equation (A3).

(A3) $$\begin{array}{cc} M_{ij} & =H(m_{i}+m_{j})\\ \; & =H(H\left(m_{i}\right)+H\left(m_{j}\right)) \end{array}$$

Root node: The hash value in the root node is the hash value of the root of the Merkle tree, which is also stored in the block header.
